# Radiographic Detection Rate of Distal Surface Caries in the Mandibular Second Molar in Populations with Different Third Molar Management Strategies: A Multicenter Study

**DOI:** 10.3390/jcm13061656

**Published:** 2024-03-14

**Authors:** Verena Toedtling, Elena-Cristina Marcov, Narcis Marcov, Dana Bodnar, Mina W. A. Hayawi, Tim Forouzanfar, Henk S. Brand

**Affiliations:** 1 Department of Oral and Maxillofacial Surgery/Oral Pathology, Amsterdam University Medical Centre/Academic Centre for Dentistry Amsterdam (ACTA), 1081 HV Amsterdam, The Netherlands; 2 Department of Operative Dentistry, Faculty of Dentistry, Carol Davila University of Medicine and Pharmacy, 050474 Bucharest, Romania; elena.marcov@umfcd.ro (E.-C.M.); narcis.marcov@umfcd.ro (N.M.); dana.bodnar@umfcd.ro (D.B.); 3 Department of Oral Biochemistry, Academic Centre for Dentistry Amsterdam (ACTA), Faculty of Dentistry, University of Amsterdam, 1081 LA Amsterdam, The Netherlands; mina-22-12@hotmail.com (M.W.A.H.); h.brand@acta.nl (H.S.B.)

**Keywords:** distal surface caries, third molar, third molar guidelines, preventative removal, interceptive treatment, third molar retention

## Abstract

**Background:** Distal surface caries (DSC) has been associated with partially erupted impacted third molars. The purpose of this study was to compare the rates of DSC between populations that had undergone different third molar management strategies. **Methods**: Radiographs that had been taken during routine examinations of 1012, 251 and 250 patients in Manchester, Bucharest and Amsterdam, respectively, were evaluated. The following parameters were assessed: the state of the distal surface in the second mandibular molar, loss of periodontal support, impaction type of the third molar, contact point localization, and patients’ genders, ages and their cumulative history of dental health. **Results**: The rate of DSC in the second mandibular molar was 63.9%, 19.9% and 26.0% in the Manchester, Bucharest and Amsterdam populations, respectively. A loss of lamina dura of ≥2 mm, increased percentages of decayed, missing or filled teeth and male gender were risk factors in all three populations. All assessed parameters apart from the site of the mandible reached statistical significance in the Manchester sample (*p* < 0.001). The DSC rate was cumulative with increasing age in the Manchester population, in which third molars were strategically retained. **Conclusions**: The UK population, treated according to strict guidelines that limit the removal of third molars, had a statistically significant higher DSC prevalence rate (*p* < 0.001) than the Romanian or Dutch populations. The active surgical management of mandibular third molars seems to have the potential to reduce the DSC rate in the adjacent second molar.

## 1. Introduction

The worldwide reported prevalence of third molar impaction across different morphological and demographic subgroups is 24.4%. Impactions occur more frequently in the mandible in comparison to the maxilla, and mesioangular impaction is the most frequently seen orientation (42%) of the lower third molar overall. Vertical and distal angulations comprise 26% and 12% of all impactions respectively; horizontal angulation is reported to be the least common impaction type (11%) [1]. The literature contains many examples of mesial and horizontal angulations that have been associated with caries on the distal aspect of the adjacent second molar and overall poor outcomes [2,3]. An in-depth analysis of studies into the prevalence rate of second molar distal surface caries (DSC) found that one in four patients who had been referred for assessment of a third molar showed evidence of DSC in mandibular second molars [3]. Clinical scientists have suggested that prophylactic or interceptive removal of the impacted third molar is the remedy for this problem, but global debates regarding the appropriateness of such treatment have been ongoing for many years [4,5]. Currently, there are two main strategies to manage the impacted third molars. One involves the deliberate retention of the impacted third molar unless symptoms or signs of pathology such as DSC develop. This is termed the non-intervention or third molar retention strategy. The other involves the removal of the impacted third molar prior to the development of symptoms and signs of pathoses. This is referred to as prophylactic or interceptive removal. Although this surgery is often necessary for prophylaxis of DSC, these procedures are not exempt from possible post-operative sequelae such as facial edema and neurosensory deficit of the mandibular nerve [6,7]. However, traditional surgical techniques and approaches have been modified and post-operative outcomes of patients are much improved as a result [8]. We believe that study populations in nations where third molars are removed preventatively should be compared with those in countries in which third molars are deliberately retained. This should provide evidence of whether the preventative removal of third molars prevents DSC in second mandibular molars that are adjacent to the impacted and partially erupted mandibular third molars.

Therefore, the purpose of this study was to investigate the rate of DSC in mandibular second molars that were adjacent to the impacted and partially erupted mandibular third molars through the examination of consecutive radiographs of patients who had attended dental hospitals or clinics for routine dental examinations. It was a collaborative study that involved the Carol Davila University of Medicine and Pharmacy in Bucharest, Romania; the University Dental Hospital of Manchester in the UK, which comes under the Manchester University National Health Service (NHS) Foundation Trust; and the Academic Centre for Dentistry Amsterdam in the Netherlands. In the UK, the guidelines issued by the National Institute for Health and Care Excellence (NICE) contain a very limited list of indications for third molar removal [4]. In contrast, in Romania and the Netherlands, the general strategy is to remove the impacted third molars prophylactically rather than wait until they are symptomatic or show signs of pathology. This approach, which is more flexible than that taken in the UK, leads to prompt removal of the impacted third molars with an emphasis on the prevention of third molar-related pathologies such as DSC [9]. Therefore, we hypothesized that the DSC detection rate would be lower among the Romanian and Dutch populations compared with that of the UK and, as a consequence, a difference in the risk factors associated with DSC. Therefore, the purpose of this retrospective, observational study was to determine the DSC rate in populations that had received an element of preventative care compared with a population that had been subject to a strict non-intervention strategy with regard to third molar removal surgery. Our secondary objective was to correlate our findings on the DSC rate with the orientation of the third molar impaction, contact point localization and the level of periodontal support determined by the loss of the lamina dura of the adjacent second molar, patient demographics such as age, gender and the site of the mandible involved as well as the patients’ decayed, missing and filled tooth score (DMFT), a summary measure of past dental disease experience.

## 2. Materials and Methods

The study conformed to all the relevant legal requirements including good clinical practice, the UK policy framework for health and social care research (2017) and the principles of the Declaration of Helsinki. Ethical approval was obtained from Health and Care Research Wales (ref: 20WM/0008), the UK’s Health Research Authority (HRA) and the West Midlands–Solihull Research Ethics Committee. Approval was also gained from the London committee of the HRA’s Confidentiality Advisory Group (ref: 20/CAG/0050). Ethical approval and equivalent study permission were obtained from the Scientific Research Ethics Commission of Carol Davila University of Medicine and Pharmacy in Bucharest, Romania (code PO-35-F-03, nr. 8823/01.04.2022), and the internal Ethical Review Board of the Academic Centre for Dentistry Amsterdam (ref: 2021-60997). This study is part of a PhD thesis [10].

The study sample was retrospective in nature and composed of consecutive patients who had presented to these three dental hospitals, which are in three countries in Europe. The UK sample comprised patients who had attended the Manchester University NHS (National Health Service) Foundation Trust, University Dental Hospital, Manchester, UK, who had attended for routine examinations and who had undergone bitewing and periapical radiography. The investigators also had access to panoramic radiographs that had been taken for some of the included patients. The Romanian sample comprised patients who had self-referred to the Carol Davila Dental Hospital and had attended almost exclusively for private dental check-ups. For these patients, a combination of bitewing and periapical radiographs were available. Several panoramic radiographs had also been taken; these formed part of the locally provided oral assessment and dental check-up. The Netherlands patients had attended dental appointments for routine examination, mandibular third molar assessment or caries screening, and they had bitewing, periapical or panoramic radiographs taken. The researchers were calibrated before making assessments.

The investigators at the three centers accessed and assessed the previously taken radiographs electronically via the use of a picture archiving and communication system (PACS), and they re-evaluated specific characteristics. Radiographs of patients ≥ 25 years of age who had second mandibular molars adjacent to impacted or partially erupted mandibular third molars were investigated. We only included radiographs that had been taken after 31 January 2012. Fully erupted and functional third molars were excluded from the study. Only excellent and diagnostically acceptable (‘A’) images were considered [11]. Images with positioning errors and artefacts such as severe cervical burn-out or other issues that obscured the area of interest were excluded. Images that showed second molars with extensive restorations or full coverage crowns were excluded, as were those second molars which were severely decayed. In cases in which both the left and right sides of the mandible of the same patient met our inclusion criteria, one radiographic image was selected through the tossing of a coin. We excluded all images with a head outcome from the study. The application of these inclusion and exclusion criteria resulted in a final study population that comprised 1012 patients in Manchester, 251 patients in Bucharest and 250 in Amsterdam.

**Figure 1:**
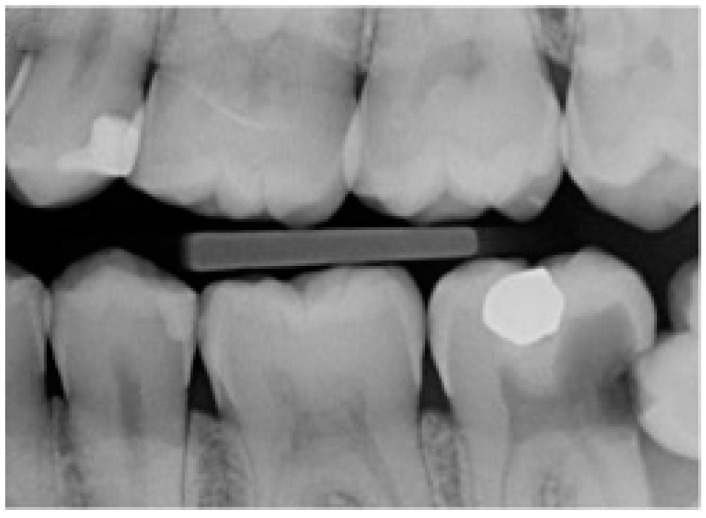
Left bitewing radiograph of a partially erupted and impacted mandibular third molar.

**Figure 2:**
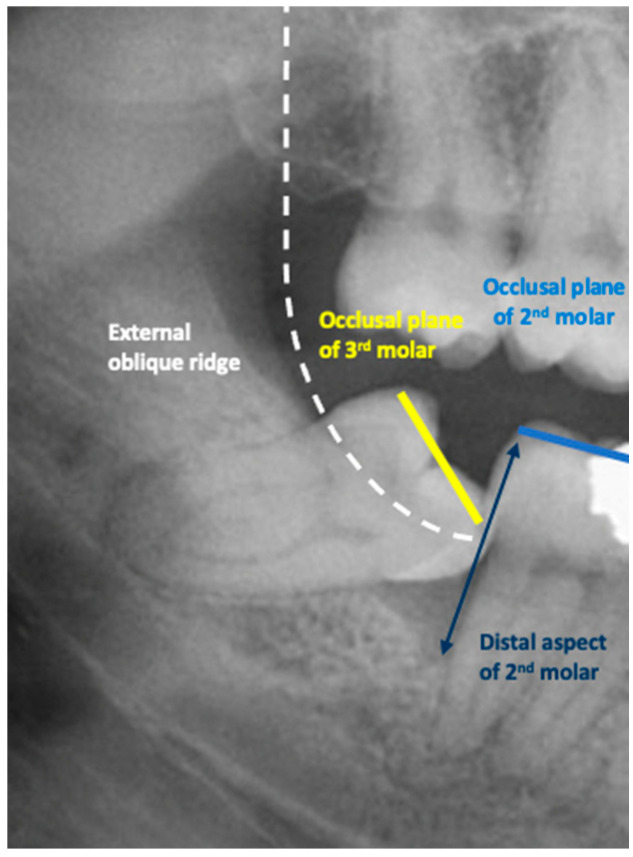
Right panoramic tomograph indicating the various anatomical landmarks required for the assessment of mandibular third molar partial eruption/emergence.

**Figure 3:**
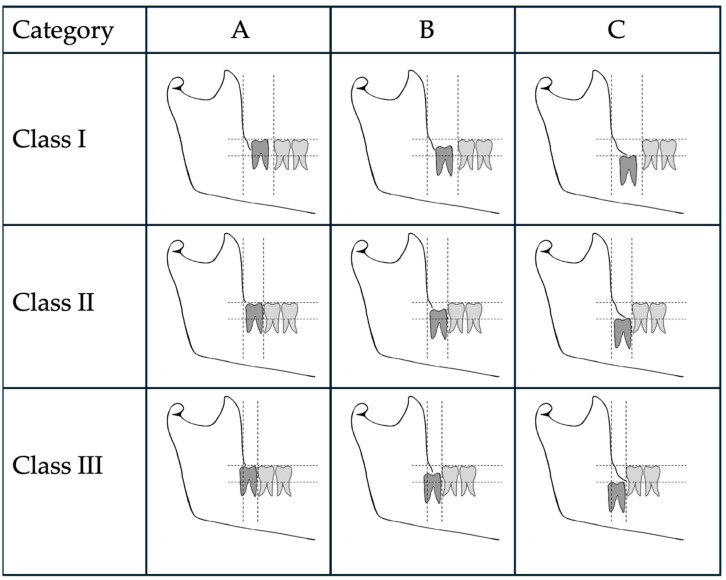
Diagram of the classification used to analyze the ratio of second to third molars to determine partial eruption (modified Pell and Gregory classification) [13].

In this study, partial emergence or partial eruption of the mandibular third molar was determined by assessing the position of the third molar in relation to that of the adjacent mandibular second molar. Figure 1 illustrates a partially erupted mandibular and impacted third molar as seen on a bitewing radiograph, while Figure 2 illustrates a panoramic tomograph of a mandibular third molar that was deemed to be partially erupted. We judged the partial eruption of the third molar from the cusp levels; the third molars were deemed to be partially emerged when one of the cusps was positioned above the occlusal plane level or above the external oblique ridge. In cases in which these anatomical landmarks were not visible on the radiograph, the cementoenamel junction (CEJ) of the adjacent second molar in relation to the position of the marginal ridge of the adjacent mandibular third molar was used to obtain information on the depth of the third molar and was used to judge its eruption status [12]. This assessment method is a modification of the original Pell and Gregory classification [13]. Classes IIIA, IB, IIB and IIIB of the original Pell and Gregory categorization were included, thus excluding fully erupted potentially functional and fully impacted third molars. This classification (Figure 3) was applied to all third molar impaction types such as mesial, horizontal, distal, vertical and transverse.

The primary outcome of interest in our study was caries on the distal aspect of the mandibular second molar (DSC). We determined a caries lesion to be present when an irregular radiolucency with irregular margins could be detected in enamel, dentine or cementum. The secondary outcomes of interest were patient demographics and oral health status. The following information was collected by the study investigators at all three study centers: patient’s gender and age (in years); side of the mandible (left or right); impaction type of the mandibular third molar according to classification by Winter; periodontal status as assessed by recording the loss of the vertical lamina dura (LD) in millimeters distally to the adjacent second molar; and the mesio-buccal cusp relationship of the mandibular third molar with the CEJ of the second molar. These data were initially recorded on a standard Excel spreadsheet (Version 14.0.4760.1000). We adapted the traditionally used index of DMFT for use in our study according to the appearance of the total number of such teeth on each radiograph. This was an adapted version and we called it modified radiographic DMFT (mDMFT-R). To calculate the mDMFT-R score, we used the following formula: DMFT count × 100/tooth count (tooth crowns fully visible on each radiograph) [12].

The radiographs were viewed and assessed in each center by one investigator to avoid inter-examiner bias. In addition, prior to data collection, a test and checks were performed in order to provide comments and feedback on the protocol and as an aid in the exchange of ideas. Multiple calibration meetings were held via Zoom and discussions about data collection methods, standards and approaches were assessed in order to standardize them in all three centers. A pilot was performed before the start of the study to assess the work and data collection flow in all centers. The entire data set of the mandibular second molar characteristics, such as the occurrence of DSC and LD loss of ≥2 mm, was reassessed in each center by a second observer and any disagreements were resolved by reaching consensus. To analyze the intra-observer agreement, 10% of all cases were randomly selected (102 from Manchester and 26 each from Bucharest and Amsterdam), reassessed and subsequently re-recorded by the same observer in each case at least six weeks apart.

### Statistical Analysis

The statistical analysis was performed through the use of the International Business Machines Statistical Package for the Social Sciences (IBM SPSS) software, version 26.0, for Macintosh (MAC release 26.0.0.0, 64-bit edition). The inter- and intra-observer agreement regarding the measurement of the radiographic findings, loss of attachment and DSC was analyzed with Cohen’s K test. An agreement of 0.75 to 1.00 was considered excellent; 0.60 to 0.74 good; 0.40 to 0.59 moderate; and less than 0.40 poor. The association between the presence of DSC in the mandibular second molar and the oral health, radiographic and demographic variables were analyzed through the application of Pearson’s chi-square independence test. Analysis of Variance (ANOVA) was used to compare the means of the three groups, followed by *t*-tests as post-hoc procedures when appropriate. All significance levels were set to a *p*-value of <0.05.

## 3. Results

**Figure 4:**
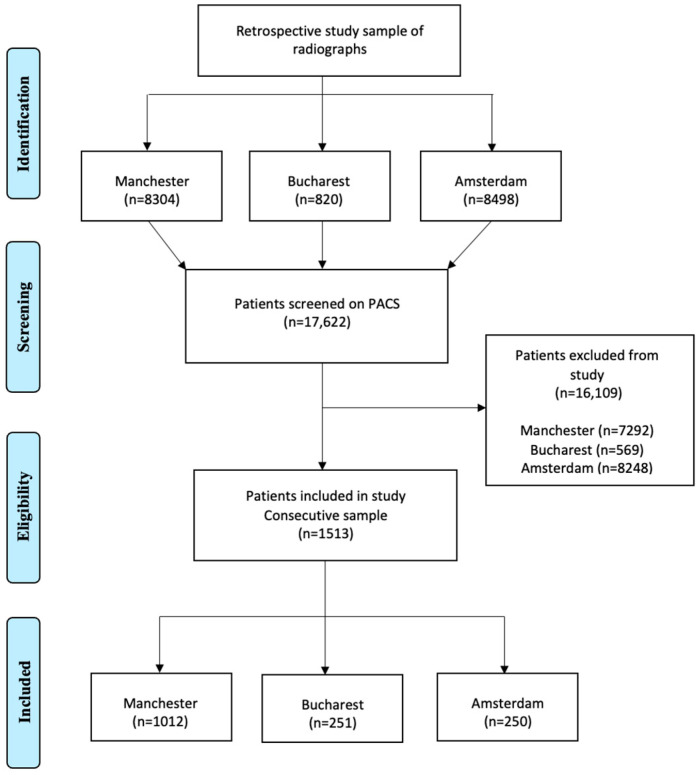
Flow chart of patients included in the multicenter study.

**Figure 5:**
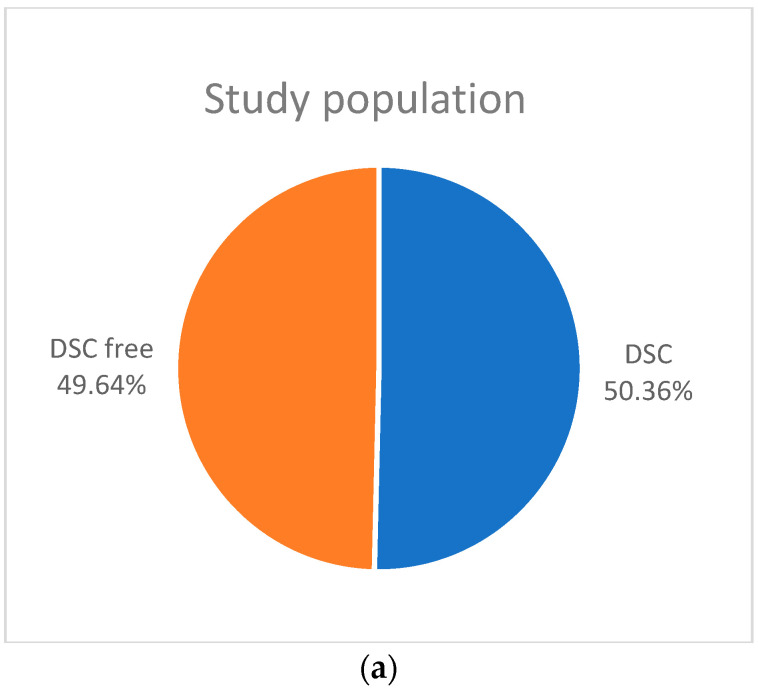

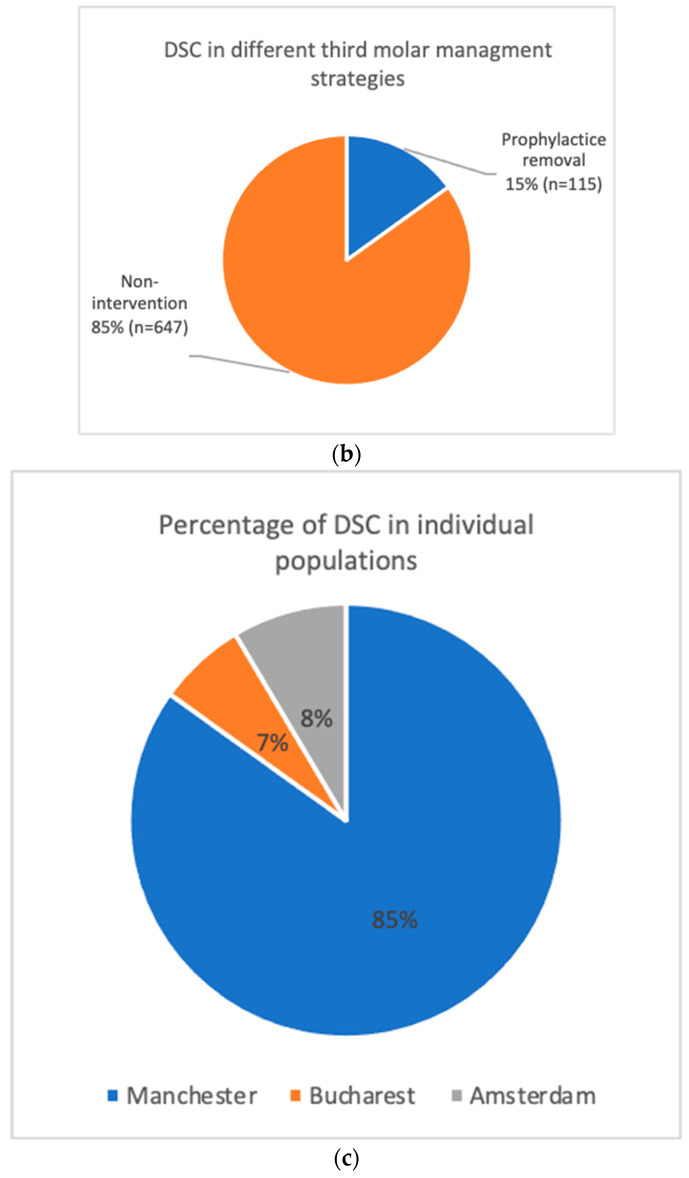
Percentages of DSC in the total study population (**a**); different third molar management strategies (**b**); and each individual population (**c**).

From the Manchester patients, a total of 8304 radiographs were viewed on PACS and 1012 patients (12.18%) were included in the study. The level of agreement between both observers for DSC and vertical loss of LD was excellent (κ = 0.776; *p* < 0.001). There was also excellent intra-observer reliability of both observers (κ of observer A = 0.812; κ of observer B = 0.797; *p* < 0.001). A total of 820 radiographs that had been taken of patients in Bucharest were viewed on PACS and 251 patients (30.61%) were included in the study. The level of agreement between both observers for DSC and vertical loss of LD was excellent too (κ = 0.752; *p* < 0.001), as was the intra-observer reliability of both observers (κ of observer A = 0.877; κ of observer B = 0.759; *p* < 0.001). In Amsterdam, 8498 patient records were assessed and 250 radiographs of patients were included (2.94%). The inter-observer reliability between both observers for DSC and vertical loss of LD was good (κ = 0.715; *p* < 0.0001) and the intra-observer reliability of both observers was excellent (κ of observer A = 0.793; κ of observer B = 0.880; *p* < 0.0001) Figure 4 presents the patient selection and Figure 5a–c illustrate the proportion of DSC in the study population.

Table 1 shows a summary of the study variables for the entire sample. In the Manchester sample, which had been managed through a third molar retention/non-intervention strategy, 647 of the 1012 patients were affected by DSC and this resulted in a DSC rate of 63.9%. Most of the affected mandibular third molars were situated on the left side of the mandible (52.6%, n = 532). The female/male gender ratio was 1:1.3 and the mean age was 37 years. Mesial impaction was most frequently observed (59.2%, n = 599); the second most frequent impaction was horizontal (20.1%, n = 203). The vast majority of the third molars showed a molar-to-molar contact point below the mesio-buccal (MB) cusp position of the adjacent third molar in relation to the CEJ of the second molar (83.6%, n = 846). 86.4% of patients from the whole sample (n = 874) had ≥2 mm loss of LD on the distal aspect of the mandibular second molar. The mean mDMFT-R of the Manchester sample was close to 48%.

In the Bucharest sample, which had been treated with a third molar preventative management strategy, 50 of the 251 patients studied were affected by DSC, resulting in a significantly lower rate of DSC (19.9%) compared with the Manchester population. Most of the affected third molars were located on the left side of the mandible (52.6%, n = 132). The female/male ratio was 1:1.3 and the mean age was 38 years. The most common direction of impaction of the third molars was mesial (42.6%, n = 107), followed by vertical (42.2%, n = 106). The vast majority of the third molars had a molar-to-molar contact point with the MB cusp below the third molar in relation to the CEJ of the second molar (79.3%, n = 199). In this sample, 84.9% of patients (n = 213) had ≥2 mm vertical loss of LD on the distal surface of the second molar. The mean mDMFT-R of the entire Bucharest sample (28%) was significantly lower than that of the Manchester sample.

In the Amsterdam sample, which had also been treated with a third molar preventative management strategy, 65 of the 250 patients were diagnosed with DSC, resulting in a DSC rate of 26%. This figure was not significantly different from that for the Bucharest population, but significantly lower than that for the Manchester sample. Most of the affected third molars were on the left mandible (59.6%, n = 149). The female/male ratio was 1:1.02 and the mean age was 33 years. The most common form of impaction of the third molars was mesial (57.2%, n = 143). The vast majority of the mandibular third molars had a molar-to-molar contact point with the MB cusp position below the third molar in relation to the second molar and its CEJ (74.0%, n = 185). Among this sample, 64.8% of patients (n = 162) had ≥2 mm loss of LD on the distal surface of the mandibular second molar. The mean mDMFT-R of the Amsterdam (32%) was lower than that of the Bucharest sample.

**Figure 6:**
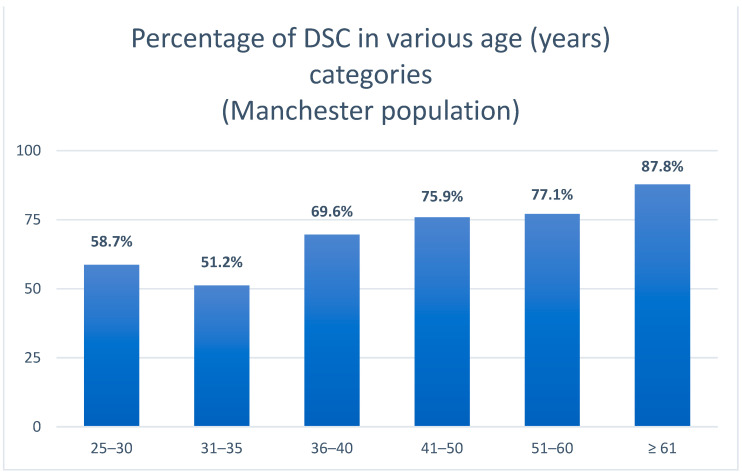
The increase in DSC with age.

Table 2 lists the clinical, demographic and oral health characteristics of the patients from Manchester who had DSC in mandibular second molars that were adjacent to the impacted third molars. Application of the Pearson chi-square independence test indicated that all variables, with the exception of the side of the mandible on which the teeth were situated, were associated significantly with the occurrence of DSC in second mandibular molars. Significantly more male patients were affected by DSC (68.2%) than were female patients (58.3%) and there was a statistically significant increase (*p* < 0.001) in the DSC rate with increasing age. DSC in the various age groups is illustrated in Figure 6. Among the different angulations of lower third molars, mesial inclination was related to the highest rate of DSC in the second mandibular molar (78.3%), followed by horizontal inclination (55.7%). DSC was observed less in patients in whom the contact point was above the third mandibular molar, compared with those patients in whom the contact point was at or below the CEJ of the second molar. DSC was significantly more frequently spotted in patients with vertical loss of LD of ≥2 mm (71.4%) and the occurrence of DSC was related to increased mDMFT-R percentages.

Table 3 shows the clinical, demographic and oral health characteristics of the patients from Bucharest who had DSC in mandibular second molars that were adjacent to the impacted third molars. In this study group, the detection rate of DSC was not related to the side of the mandible on which the second molar was situated. More male patients (22.4%) were affected than female patients (16.7%), and DSC prevalence percentages were found to remain similar with increasing age. Among the different types of impaction angulations of lower third molars, mesial impactions showed the highest rate of DSC in the second mandibular molar (24.3%), closely followed by distal impactions (23.3%). DSC was almost equally observed in the patients in whom the contact point was below (20.1%) or at (19.2%) the cusp position of the third mandibular molars. DSC was observed more frequently in patients with vertical loss of LD of ≥2 mm (21.1%) and was related to increased mDMFT-R percentages. With the exception of mDMFT-R, none of the relationships reached statistical significance.

The clinical, demographic and oral health characteristics of patients from Amsterdam who had DSC in mandibular second molars that were adjacent to the impacted third molars are shown in Table 4. In this study population, the rate of DSC was not related to the side of the mandible on which the second molar was situated, nor to gender or age. Significant differences were observed in DSC rate among the different angulations of lower third molars, with horizontal impactions showing the highest rate of DSC in the second molar (42.4%), followed by mesially impacted mandibular third molars (29.4%). The difference in DSC rate between patients in whom the contact point was below (29.2%) or at (16.9%) the cusp position of the third mandibular molars almost reached statistical significance (*p* = 0.052). Patients who had loss of LD of ≥2 mm (34.6%) were significantly more frequently to have DSC, and DSC was also significantly related to increased mDMFT-R percentages.

## 4. Discussion

Should asymptomatic, disease-free, impacted third molars be removed prophylactically because they may cause local disease? A Cochrane systematic review of observational studies was performed in 2020 with the aim of answering this globally debated research question [14]. DSC was one of the long-term outcomes that was assessed in this review. However, the review included only one relevant study on DSC which had a high risk of bias, conducted among an insured North American population and which could not identify clear evidence for or against the removal of third molars to prevent DSC. The aim of the present observational study, although retrospective and cross-sectional in nature, was to compare the rate of DSC in a European population, to which a non-intervention strategy with regard to third molar surgery had been applied, with the DSC detection rate in two populations that have been treated preventatively. Data were collected regarding patients in three European countries who had undergone radiography as part of routine dental check-ups. Various clinical characteristics, such as the impaction type and contact point localization of the mandibular third molar, periodontal support of the adjacent second molar, a summary measure of past dental disease experience and patient demographics, were compared. We hypothesized that DSC would be more prevalent in people who lived in nations in which third molars were retained. We also hypothesized that knowledge of epidemiological detection rate for diverse populations would aid in the understanding of risk factors for the development of DSC and could be used to identify differences between groups of patients who had been exposed to different third molar treatment strategies.

In this multicenter study, dental surgeons in all three centers detected a rate of DSC in the second molar that was adjacent to the impacted and partially erupted mandibular third molars among populations that accessed general dental care. We found that the rate in the Manchester population was statistically significantly higher than in those in Bucharest and Amsterdam (64% versus 20% and 26%, respectively) (*p* < 0.001). The lower rate shown in the Bucharest sample might partly be because most patients had undergone panoramic radiographs as part of their routine dental assessment, while the Manchester data were composed of consecutive intra-oral radiographs from an archive. Intra-oral radiographs show much greater sensitivity in the detection of caries in comparison with extra-oral radiographs [15], so DSC may have been observed more clearly in the Manchester data than in the Bucharest images. However, the rate among the Amsterdam cohort was almost exclusively calculated from intra-oral radiographs and was similarly lower than that of the Bucharest group. We could not find a multicenter study using intra-oral radiographs in the literature to make a comparison to our study finding, but we believe that this finding suggested that the higher rate of DSC in Manchester was not only due to the use of different types of radiographs to determine the rate of DSC.

The Bucharest study population showed fewer mesial and horizontally impacted third molars than the Manchester and Amsterdam samples. Allen et al. also reported that both types of impactions have been identified in the literature as those most frequently associated with DSC in cases in which third molars are retained [16]. This may have contributed to the fact that the lowest DSC rates were found in the Bucharest population. Furthermore, the patients seen in Bucharest and Amsterdam had lower average mDMFT-R percentage scores than those in Manchester, which indicated that they had, on average, better dental health than the patients from Manchester. However, we believe that these reasons do not entirely explain the 2.5 to 3.2-fold higher DSC rate in the Manchester study sample. While UK dentists adhere strictly to NICE guidelines, clinicians in Romania and the Netherlands discuss the consequences of third molar retention and the risk of DSC with patients, and in most cases, these discussions result in proactive third molar removal [9]. Therefore, the high rate of DSC occurrence in the Manchester population could in part be related to the long-term retention of third molars due to strict removal guidance [17], although a causal link has not been established.

The literature describes a strong association between DSC development and mesially impacted third molars that are adjacent to mandibular second molars [18]. This strong association was also found in our study, and in the Manchester and Bucharest populations, third molars with mesial angulation were indeed associated with the greatest rate of DSC in the second molar. However, in the Amsterdam population, DSC was observed more often in third molars that were impacted horizontally than in those angled mesially. However, horizontal impaction was the second most frequent angulation that was associated with the presence of DSC in the Manchester group; these findings were in line with previous reports and studies [18,19]. In the Bucharest population, the second most frequent third molar angulation associated with the presence of DSC was vertical impaction, which was not in line with the Manchester or Amsterdam results. We speculate that this could be because the third molars had been preventatively removed from the Bucharest subjects, which would have affected the distribution of different impaction cases in this population. In addition to this point, Shepherd and Brickley stated in 1994 that the impacted third molars which caused the most pathological risk were of vertical type and that patients with this type of impaction presented most frequently for removal. Interestingly, this observation was also reported and documented in the literature during a time when prophylactic third molar removal was routinely performed in the UK pre-NICE guidance, and may explain the difference in the case mix observed [20].

Another anatomical variation that was associated with the occurrence of DSC in our study in all three populations was the region of the molar-to-molar contact point and contact below the CEJ. These factors were associated with a much greater risk of DSC occurrence compared with third molars that had no contact, or those that were at or above the CEJ of the adjacent second molar. Özeç et al. reported very similar findings in their research. They also investigated the molar-to-molar contact as well as CEJ distance and explained that this is the distance between the mesial CEJ of the third molar and the distal CEJ of the second molar (an arbitrary line through the embrasure). They showed the more the third molar was tilted mesially, the more the CEJ of the third molar moved distally, and subsequently the CEJ distance would lengthen and the associated embrasure would become larger. This provides greater potential for plaque stagnation and increases the likelihood of DSC [21]. In addition to this, Chen et al. revealed a linear correlation between third molar mesial angulation and CEJ distance, and they reported that DSC prevalence is increased in the presence of third molars that show contact points at or below the second mandibular molar CEJ [22].

Our study also showed that radiographic evidence of a vertical loss of LD ≥2 mm on the distal aspect of the second molars were much more likely to be associated with DSC than were second molars with a loss of LD <2 mm. Consequently, we consider that the loss of LD of ≥2 mm indicates a mild loss of attachment and the development of periodontal disease, a precursor to DSC. This was evident in all three of our study populations. We are not aware of any other study population that has been assessed for LD loss as an indicator of DSC development, so we suggest that this parameter should be included in future research on DSC and this would provide a comparison.

The study populations in Manchester and Bucharest were of similar mean ages, with the mean age of the Amsterdam population being a few years younger. Within the Manchester population, there was almost a five-year difference between the average age of those with DSC (38.1 years) and those without (33.9 years), while in the Bucharest and Amsterdam populations, no significant difference in age was observed between patients with and without DSC. Also, among the Manchester patients, DSC rate markedly increased with age, while in the Bucharest and Amsterdam samples, the level of DSC did not. Such a finding and characteristics would be unusual for caries in general, as it is a disease that builds over a person’s lifetime in the presence of substrate and bacteria. However, this is another indication that the preventative removal of third molars may prevent age-related increases in rates of DSC. This specific finding has not previously been documented in the literature; however, Renton at al. have demonstrated that third molars are removed on average at an older age following the introducing of the NICE guidelines in the UK [23].

Relatively small but statistically significant differences were observed regarding the mDMFT-R percentage scores. The mean mDMFT-R percentage was 19.1 percentage points less in the Bucharest sample and 16.0 percentage points less in the Amsterdam sample than in the Manchester sample. This suggests that the Bucharest and Amsterdam samples had better dental health to a statistically significant degree than the Manchester sample. Clinically, this would translate as the Bucharest and Amsterdam patients having on average approximately 1.3 to 1.5 times fewer DMFT. Our findings do not fall in line with previous research results from McArdle and McDonald (2019), who reported lower DMFT scores among patients with DSC when compared with those of a regularly surveyed general population [18]. In the present study, patients from the Manchester sample with DSC had a mean mDMFT-R that was statistically significantly higher in comparison with patients from Manchester free of DSC, and the mean mDMFT-R was statistically significantly lower in the Bucharest and Amsterdam DSC populations than in the Manchester DSC population. The clinical significance of this observation is limited, firstly because the differences in mDMFT-R were relatively small and it was unclear how this could be clinically compared, and secondly because the patients with DSC in the Manchester sample were on average four years older than those without, and it is universally known that caries experience and accompanying DMFT scores increase with age.

A previous study designed to detect the DSC rate by means of Cone Beam Computer Tomography (CBCT) scan suggested that the prevalence of DSC in the second molars of men is higher than that in women [22]. This was also observed in all three population samples in our study. The literature consistently documents that female patients exhibit better oral health behaviors and higher levels of oral hygiene than men [24,25].

The results of the present study confirmed our hypothesis in part, as DSC was found to be significantly more prevalent among patients who had been subject to the non-intervention strategy in Manchester than among those who had access to and were given the possibility to have preventative care in Bucharest and Amsterdam. It should not be a surprise that employing different third molar management strategies results in different outcomes for our patients. However, it must be understood that other factors apart from differences in national recommendations/guidelines on third molar management strategies may have contributed to the differences among the various populations. For example, skill levels amongst clinical teams, resource allocation and remuneration systems for dental and health care or patients’ socioeconomic factors may have secondarily affected the various DSC rate that were observed.

One of the limitations of our study was that our study design was cross-sectional in nature; consequently, it gives no indications of the sequence of events and it is not possible to infer causality. Conducting a longitudinal study or randomized controlled clinical trial investigating the long-term effects such as DSC of asymptomatic third molar retention vs. removal in a representative sample are highly desirable. However, the advantages of the present study were the multicenter approach and the relatively large study populations that could be drawn from each participating research center with calibrated teams. Even though this study was retrospective, we designed it in such a way that dental health and oral hygiene data that were drawn from it could be assessed, as these factors are important in caries detection rate. A further limitation was that there was no clinical or histological verification of the presence of caries, and potential radiological artefacts such as burn-out and the March band effect, or even root resorption, could not be excluded in all cases. We assumed that a radiolucent area on the distal aspect of the second molars next to an impacted third molar was due to caries, which is a valid assumption, but confirming this with a clinical diagnosis would be better. Therefore, we recommend that future research and studies should also include clinical verification of the accuracy of diagnoses. In addition to this, the socioeconomic status of individual patients could not be compared among the patients of the three study populations. Therefore, future studies on this research topic should include assessments of patients’ socioeconomic backgrounds via deprivation categories from, for example, postal codes to explore whether there is a correlation or association between socioeconomics and DSC development in different populations. Although there are a number of limitations, this multicenter study provides insight into the epidemiology of DSC in the second molars of populations that access general dental care in the UK, Romania and the Netherlands, and thus contributes to research in this field. We envisage that third molar research on DSC has worldwide significance for millions of patients because the indications for third molar removal surgery have been debated on a global level for many years. Our findings show that the retention of third molars is strongly associated with DSC and patients suffer harm as a result. We believe that this novel information concerning the management of third molars is likely to encourage worldwide discussions amongst oral and maxillofacial surgeons, general dental practitioners, clinical scientists and researchers and initiate larger-scale longitudinal studies, providing greater precision and a higher level of evidence which has the potential to impact patient care, inform policy and ultimately improve the health of our patients.

## 5. Conclusions

The population that was governed by restrictive guidelines regarding third molar removal had a much greater DSC rate than did the populations that could undergo preventative third molar removal. In the former population, DSC accumulated significantly with age; this was not found in the latter populations. Although the epidemiological data on DSC are limited, these results support the assumption that retention of third molars is associated with an increased risk of second molar pathology such as DSC. Ultimately, to better determine the impact of third molar guidelines on DSC occurrence, future studies of populations with similar socioeconomic status in nations that practice preventative or interceptive third molar removal are warranted. Such studies should be of a higher level of evidence, such as case-control or cohort study design, and include the collection of clinical data such as lesion color, texture, plaque index, probing depths, bacterial composition and the extent of caries in addition to the radiographic data, to provide evidence regarding whether or not preventative third molar removal prevents the development of DSC in second mandibular molars adjacent to the impacted or partially erupted third molars.

## Figures and Tables

**Table 1:** Summary of study variables for the samples from the three research centers.

Population	Manchester (n = 1012)	Bucharest (n = 251)	Amsterdam (n = 250)
Third Molar Strategy	Retention Strategy	Preventative Removal	Preventative Removal
Characteristics	n (% of Total)	n (% of Total)	n (% of Total)
Rate of DSC	647 (63.9)	50 (19.9)	65 (26.0)
Side of mandible			
Right	480 (47.4)	119 (47.4)	101 (40.4)
Left	532 (52.6)	132 (52.6)	149 (59.6)
Gender			
Female	434 (42.9)	108 (43.0)	126 (50.4)
Male	578 (57.1)	143 (57.0)	124 (49.6)
Age (years)			
Mean age (years) ± standard deviation (SD)	36.6 ± 11.1	37.5 ± 9.9	32.7 ± 9.7
25–30	402 (39.4)	59 (23.5)	138 (55.2)
31–35	205 (20.3)	81 (32.3)	56 (22.4)
36–40	115 (11.4)	47 (18.7)	18 (7.2)
41–50	166 (16.4)	40 (15.9)	21 (8.4)
51–60	83 (8.2)	15 (6.0)	13 (5.2)
≥61	41 (4.1)	9 (3.6)	4 (1.6)
Orientation of third molar impaction			
Mesial	599 (59.2)	107 (42.6)	143 (57.2)
Horizontal	203 (20.1)	8 (3.2)	33 (13.2)
Vertical	98 (9.7)	106 (42.2)	21 (8.4)
Distal	109 (10.8)	30 (12.0)	53 (21.2)
Transverse	3 (0.3)	0 (0)	0 (0)
Contact point localization: MB cusp position			
Above	26 (2.6)	0 (0)	0 (0)
At	110 (10.9)	52 (20.7)	65 (26.0)
Below	846 (83.6)	199 (79.3)	185 (74.0)
No contact	30 (3.0)	0 (0)	0 (0)
Loss of LD			
<2 mm	138 (13.6)	38 (15.1)	88 (35.2)
≥2 mm	874 (86.4)	213 (84.9)	162 (64.8)
Mean mDMFT-R (%) ± SD	47.5 ± 28.2	28.4 ± 21.7	31.5 ± 25.0
0	73 (7.2)	6 (2.4)	48 (19.2)
1–15	80 (7.9)	66 (26.3)	33 (13.2)
16–30	174 (17.2)	78 (31.1)	54 (21.6)
31–45	179 (17.7)	69 (27.5)	45 (18.0)
46–60	167 (16.6)	16 (6.4)	34 (13.6)
61–75	173 (17.1)	13 (5.2)	21 (8.4)
≥76	166 (16.4)	3 (1.1)	15 (6.0)

MB cusp, mesio-buccal cusp; LD, lamina dura; mDMFT-R, modified decayed, missing or filled tooth index applied to radiographs.

**Table 2:** Radiographic detention rate of DSC in second molars adjacent to the impacted mandibular third molars and its relation to demographic, clinical and oral health characteristics in patients from Manchester, which has a third molar retention strategy.

	Total (n = 1012)	Presence of DSC		*p*-Value
Characteristics	n (%)	Yes (%)	No (%)	
Side of mandible				0.883
Right	480 (47.4)	308 (64.2)	172 (35.8)	
Left	532 (52.6)	339 (63.7)	193 (36.3)	
Gender				0.001 *
Female	434 (42.9)	253 (58.3)	181 (41.7)	
Male	578 (57.1)	394 (68.2)	184 (31.8)	
Age (years)				<0.001 *
Mean age (years) ± SD	36.6 ± 11.1	38.1 ± 11.9	33.9 ± 8.9	<0.001 *
25–30	402 (39.4)	236 (58.7)	166 (41.3)	
31–35	205 (20.3)	105 (51.2)	100 (48.8)	
36–40	115 (11.4)	80 (69.6)	35 (30.4)	
41–50	166 (16.4)	126 (75.9)	40 (24.1)	
51–60	83 (8.2)	64 (77.1)	19 (22.9)	
≥61	41 (4.1)	36 (87.8)	5 (12.2)	
Orientation of third molar impaction				
Mesial	599 (59.2)	469 (78.3)	130 (21.7)	<0.001 *
Horizontal	203 (20.1)	113 (55.7)	90 (44.3)	
Vertical	98 (9.7)	38 (38.8)	60 (61.2)	
Distal	109 (10.8)	25 (22.9)	84 (77.1)	
Transverse	3 (0.3)	2 (66.7)	1 (33.3)	
Contact point localization: MB cusp position				
Above	26 (2.6)	9 (24.6)	17 (65.4)	<0.001 *
At	110 (10.9)	30 (27.3)	80 (72.7)	
Below	846 (83.6)	599 (70.8)	247 (29.2)	
No contact	30 (3.0)	9 (30.0)	21 (70.0)	
Loss of LD				<0.001 *
<2 mm	138 (13.6)	23 (16.7)	115 (83.3)	
≥2 mm	874 (86.4)	624 (71.4)	250 (28.6)	
mDMFT-R (%)				<0.001 *
Mean mDMFT-R (%) ± SD	47.5 ± 28.2	51.8 ± 27.7	39.9 ± 27.6	<0.001 *
0	73 (7.2)	22 (30.1)	51 (69.9)	
1–15	80 (7.9)	40 (50.0)	40 (50.0)	
16–30	174 (17.2)	111 (63.8)	63 (36.2)	
31–45	179 (17.7)	118 (65.9)	61 (43.1)	
46–60	167 (16.6)	115 (68.9)	52 (31.1)	
61–75	173 (17.1)	107 (61.8)	66 (38.2)	
≥76	166 (16.4)	134 (80.7)	32 (19.3)	

MB cusp, mesial-buccal cusp; LD, lamina dura; mDMFT-R, modified decayed, missing or filled teeth index applied to radiographs. * Statistically significant (*p* < 0.05) Pearson’s chi-square independence test between categorical variables and the *t*-test or analysis of variance (ANOVA) between different means. Table 2 in this article has been published in BMC Oral Health and is available online at https://doi.org/10.1186/s12903-023-02766-w (accessed on 9 November 2023) [12].

**Table 3:** Radiographic detection rate of DSC in second molars adjacent to the impacted mandibular third molars, and its relation to demographic, clinical and oral health characteristics in patients in Bucharest, which has a third molar preventative removal strategy.

	Total (n = 251)	Presence of DSC		*p*-Value
Characteristics	n (%)	Yes (%)	No (%)	
Side of mandible				0.136
Right	119 (47.4)	19 (16.0)	100 (84.0)	
Left	132 (52.6)	31 (23.5)	101 (76.5)	
Gender				0.262
Female	108 (43.0)	18 (16.7)	90 (83.3)	
Male	143 (57.0)	32 (22.4)	111 (77.6)	
Age (years)				0.773
Mean age (years) ± SD	37.5 ± 9.9	36.4 ± 9.8	38.1 ± 9.2	0.250
25–30	59 (23.5)	14 (22.7)	45 (76.3)	
31–35	81 (32.3)	17 (21.0)	64 (79.0)	
36–40	47 (18.7)	8 (17.0)	39 (82.0)	
41–50	40 (15.9)	8 (20.0)	32 (80.0)	
51–60	15 (6.0)	1 (6.7)	14 (93.3)	
≥61	9 (3.6)	2 (22.2)	7 (77.8)	
Orientation of third molar impaction				
Mesial	107 (42.6)	26 (24.3)	81 (75.7)	0.344
Horizontal	8 (3.2)	1 (12.5)	7 (87.5)	
Vertical	106 (42.2)	16 (15.1)	90 (84.9)	
Distal	30 (12.0)	7 (23.3)	23 (76.7)	
Transverse	0 (0)	0 (0)	0 (0)	
Contact point localization: MB cusp position				
Above	0 (0)	0 (0)	0 (0)	0.889
At	52 (20.7)	10 (19.2)	42 (80.8)	
Below	199 (79.3)	40 (20.1)	159 (79.9)	
No contact	0 (0)	0 (0)	0 (0)	
Loss of LD				0.257
<2 mm	38 (15.1)	5 (13.2)	33 (86.8)	
≥2 mm	213 (48.9)	45 (21.1)	168 (78.9)	
mDMFT-R (%)				0.103
Mean mDMFT-R (%) ± SD	28.4 ± 21.7	36.3 ± 33.35	26.3 ± 17.2	0.0034 *
0	6 (2.4)	0 (0)	6 (100)	
1–15	66 (26.3)	11 (16.7)	55 (83.3)	
16–30	78 (31.1)	11 (14.1)	67 (85.9)	
31–45	69 (27.5)	18 (26.1)	51 (73.9)	
46–60	16 (6.4)	4 (25.0)	12 (75.0)	
61–75	13 (5.2)	4 (30.8)	9 (69.2)	
≥76	3 (1.1)	2 (66.7)	1 (33.3)	

MB cusp, mesio-buccal cusp; LD, lamina dura; mDMFT-R, modified decayed, missing or filled tooth index applied to radiographs. * Statistically significant (*p* < 0.05) Pearson’s chi-square independence test between categorical variables and the *t*-test or analysis of variance (ANOVA) between different means.

**Table 4:** Radiographic detection rate of DSC in second molars adjacent to the impacted mandibular third molars and its relation to demographic, clinical and oral health characteristics in patients in Amsterdam, which has a third molar preventative removal strategy.

	Total (n = 250)	Presence of DSC		*p*-Value
Characteristics	n (%)	Yes (%)	No (%)	
Side of mandible				0.164
Right	101 (40.4)	31 (30.7)	70 (69.3)	
Left	149 (59.6)	34 (22.8)	115 (77.2)	
Gender				0.170
Female	126 (50.4)	28 (22.2)	98 (77.8)	
Male	124 (49.6)	37 (29.8)	87 (70.2)	
Age (years)				0.139
Mean age (years) ± SD	32.7 ± 9.7	33.8 ± 10.3	32.3 ± 9.4	0.757
25–30	138 (55.2)	32 (23.1)	106 (76.8)	
31–35	56 (22.4)	16 (28.6)	40 (71.4)	
36–40	18 (7.2)	5 (27.8)	13 (72.2)	
41–50	21 (8.4)	7 (33.3)	14 (66.7)	
51–60	13 (5.2)	3 (23.1)	10 (76.9)	
≥61	4 (1.6)	2 (50.0)	2 (50.0)	
Orientation of third molar impaction				0.003 *
Mesial	143 (57.2)	42 (29.4)	101 (70.6)	
Horizontal	33 (13.2)	14 (42.4)	19 (57.6)	
Vertical	21 (8.4)	1 (4.8)	20 (95.2)	
Distal	53 (21.2)	8 (15.1)	45 (84.9)	
Transverse	0 (0)	0 (0)	0 (0)	
Contact point localization: MB cusp position				0.052
Above	0 (0)	0 (0)	0 (0)	
At	65 (26.0)	11 (16.9)	54 (83.1)	
Below	185 (74.0)	54 (29.2)	131 (70.8)	
No contact	0 (0)	0 (0)	0 (0)	
Loss of LD				<0.001 *
<2 mm	88 (35.2)	9 (10.2)	79 (89.8)	
≥2 mm	162 (64.8)	56 (34.6)	106 (65.4)	
mDMFT-R (%)				<0.001 *
Mean mDMFT-R (%) ± SD	31.5 ± 25.0	39.9 ± 25.7	28.6 ± 24.1	0.035 *
0	48 (19.2)	5 (10.4)	43 (89.6)	
1–15	33 (13.2)	9 (27.3)	24 (72.7)	
16–30	54 (21.6)	13 (24.1)	41 (75.9)	
31–45	45 (18.0)	14 (31.1)	31 (68.9)	
46–60	34 (13.6)	8 (23.5)	26 (76.5)	
61–75	21 (8.4)	10 (47.6)	11 (52.4)	
≥76	15 (6.0)	6 (40.0)	9 (60.0)	

MB cusp, mesio-buccal cusp; LD, lamina dura; mDMFT-R, modified decayed, missing or filled tooth Index applied to radiographs. * Statistically significant (*p* < 0.05) Pearson’s chi-square independence test between categorical variables and the *t*-test or analysis of variance (ANOVA) between different means.

## Data Availability

The data presented in this study are available on request from the corresponding author.
